# Effects of Value Perception, Environmental Regulation and Their Interaction on the Improvement of Herdsmen’s Grassland Ecological Policy Satisfaction

**DOI:** 10.3390/ijerph18063078

**Published:** 2021-03-17

**Authors:** Mingyue Li, Pujie Zhao, Lianbei Wu, Kai Chen

**Affiliations:** School of Economics and Management, Beijing Forestry University, Beijing 100083, China; limingyue_2019@bjfu.edu.cn (M.L.); zhaopujie@bjfu.edu.cn (P.Z.); wulianbei@bjfu.edu.cn (L.W.)

**Keywords:** grassland ecology, grassland subsidy, overgrazing, environmental degradation, herdsmen’s livelihood, multiordered Logit model

## Abstract

Sustainable utilization of grassland resources was an important topic concerned by worldwide countries and regions, and ecological compensation had gradually become the main policy tool for grassland environmental management and ecological protection. This study adopted face-to-face interviews and questionnaires, and multiordered Logit model was then used to explore herdsmen’s satisfaction with Grassland Ecological Conservation Subsidy and Reward Policy (GECSRP) focusing on identifying the key factors behind it. Results showed that herdsmen were not satisfied with GECSRP on the whole, while value perception, environmental regulation and their interaction played a positive role on improving the satisfaction. Specifically, economic benefits had the strongest promotion impacts, followed by social identity in the two-dimensional variables of value perception. The guiding regulation had stronger promoting impacts, followed by the incentive regulation in the two-dimensional variables of environmental regulation. Interestingly, incentive regulation played an enhanced interaction on the influence of economic benefits and environmental value on herdsmen’s satisfaction, yet the interaction between guiding regulation and environmental value was not significant. These indicated that herdsmen paid more attention to substantial subsidies and rewards in the process of ecological livestock husbandry, and environmental regulation formulated by government had a phenomenon of “relative system failure”. Thus, the grassland ecological environment policy should be further adjusted and improved to promote the economic development of pastoral areas.

## 1. Introduction

As an important part of natural resources, grassland played an increasingly important role on promoting ecological civilization construction and economic development. However, the vicious cycle of grassland ecological environment deterioration was increasingly intensified due to frequent natural disasters and unreasonable utilization of grassland by human beings [1]. This seriously restricted the sustainable utilization and development of grassland resources. According to the statistics, about 20% of natural grassland and 73% of pasture grassland in the world had degenerated to varying degrees. For example, the vegetation coverage decreased sharply due to pasture expansion in about 70% of the grassland of the Amazon region [2] and 78% of the typical grassland in Mongolia had changed into dry or desert grassland [3]. In Australia, some punctate vegetation increased successively due to the low productivity of grassland [4], while the problems of soil and water loss were serious caused by the expansion of pastures in Switzerland [5]. Nearly 50% of grasslands in China were experiencing a decline in greenness and productivity, where the Inner Mongolia Autonomous Region was the worst [6]. The evidence above suggested that grassland resources were being severely challenged by the degeneration, and overgrazing was considered to be the main cause of this phenomenon. This could seriously affect the production and life of those who took the grassland as herdsmen’s main means of livelihood.

To effectively solve the ecological and economic problems caused by overgrazing, the grassland protection plans were issued by many countries. The UK formulated an agricultural environment plan to encourage herdsmen to give up their original grassland management model and accept government subsidies [7]. The US launched a resource conservation plan that emphasized reducing livestock costs to improve the profitability of ranches [8]. Australia implemented a reward system for grassland classification to reduce the crowding-out effect of real estate development on pasture management [6]. In 2016, China launched a new round of the Grassland Ecological Conservation Subsidy and Reward Policy (GECSRP), which gave priority to protecting the grassland ecological environment and steadily increased the herdsmen’s income through promoting the transformation of the production and management mode of animal husbandry. In 2020, the efforts were emphasized again to further promote ecological poverty alleviation on the grasslands, and it was highlighted that subsidy funds should be delivered to herdsmen timely and fully. Nevertheless, the policy effects were limited in practice, because the forbidden grazing behaviors caused by “imbalance between human and livestock” could not be effectively controlled, and the policy did not attract much support among herdsmen groups [9]. The implementation of the GECSRP was closely related to the herdsmen livelihood, and their satisfaction would affect whether they continued to participate in the plan. Therefore, how to improve herdsmen’s satisfaction with the GECSRP still needs further in-depth research.

In the existing literature, individual characteristics (e.g., education level, whether they have part-time jobs) [10], family endowment (e.g., labor force ratio, household income) [3], production characteristics (e.g., breeding scale, pasture area) [8], social environment (e.g., network relationships, environmental rewards) [11] were considered as important factors affecting herdsmen’s satisfaction. These studies enriched our understanding of herdsmen’s satisfaction from the perspective of demographic and socioeconomic characteristics, yet there was limited research exploring the satisfaction focusing on the internal perception and external environment. To fill this gap, this study would introduce value perception and environmental regulation variables, and deeply investigate their impacts on the herdsmen’s satisfaction in a unified framework. Herdsmen were the important subjects of grassland grazing activities. Their attitude could directly affect satisfaction, and value perception was a key influencing factor of attitude formation [12,13]. This indicated that herdsmen’s satisfaction was largely affected by value perception. Besides, Zhang et al. [14] found that although the subsidy standard could not make up for the extra efforts of herdsmen, such eco-friendly incentive policies still had significant impacts on their decision-making. Environmental policies were conducive to reducing resource consumption and pollutant emissions, thus enhancing subjective well-being [15], and the well-being level was closely related to the residents’ satisfaction [16]. Therefore, it could be inferred that the herdsmen’s satisfaction was also likely to be affected by government-led environmental regulations.

Based on the above considerations, the main purpose of this study was to determine the herdsmen’s satisfaction with GECSRP and identify the key influencing factors behind it. The specific objectives were as follows: (1) according to the field investigation of herdsmen in the four major pastoral areas in Inner Mongolia of China, a theoretical analysis framework of herdsmen satisfaction was constructed from the perspective of value perception and environmental regulation; (2) identifying herdsmen’s satisfaction with GECSRP, and further exploring the impacts of value perception, environmental regulation and their interaction on herdsmen’s satisfaction. The information here could provide new references for policy makers and practitioners to adjust and improve the policies to save grassland resources. The structures of this article were arranged as follows. Section 2 combed the literature on value perception and environmental regulation, and then put forward research hypotheses. Section 3 presented materials and methods, including the research areas overview and sample data sources. Meanwhile, the selection of scale variables and the construction of economic model were introduced. The results and discussion would be presented in Section 4 and Section 5, respectively. The last section mainly summarized the research conclusions and provided some policy implications for the sustainable development of grassland and animal husbandry in the research areas.

## 2. Theoretical Framework and Hypothesis Development

### 2.1. Value Perception

Value perception referred to the subjective feeling after the comprehensive tradeoff between costs and benefits in the decision-making process. It originated from the previous research on general value and environmental value [17]. Xie et al. [18] pointed out that value perception had a significant impact on customer satisfaction, and satisfaction was generally based on customers’ evaluation of actual experience and perceived experience. This had been widely used to identify human evaluation of objective things and analysis of influencing factors. In terms of specific dimensions, Sheth et al. [19] believed that value perception could be divided into five dimensions: social perception, emotional perception, functional perception, cognitive perception, and conditional perception. Sweeney and Soutar [20] developed a scale to measure value perception and simplified the initial five dimensions into three, namely functional value, social value, and emotional value. Cao and Zhao [21] divided value perception into three aspects (self-interest, altruism and ecology) to explore its effect on farmers’ intentions to reduce fertilizer application. The definition of value perception was different in different research scenarios [22]. In the context of Chinese society, this study drew on the existing research results and the actual situation of the investigated pastoral areas, and divided the herdsmen’s value perception into three dimensions, namely economic benefits, environmental value and social identity. Hence the following hypothesis was proposed:

**Hypothesis 1 (H1).**  *Value perception had positive effects on herdsmen’s GECSRP satisfaction*.

Perception in the economic aspect had been identified as an important factor in constructing the dimension of value perception. Parasuraman and Grewal [23] concluded that “consumers’ price perception was the key factor affecting their satisfaction”. This supported the inclusion of economic benefits into the specific dimension of value perception, because price was a typical economic embodiment in the field of consumption research [12]. In terms of GECSRP, herdsmen were the direct perceivers and beneficiaries of the policy subsidies and incentives. Compared with the herdsmen’s original grazing mode, the ecological animal husbandry advocated by the policy often led to higher costs for herdsmen. Therefore, the perception of the overall economic benefits from the implementation of ecological animal husbandry should be involved in the scope of the needs of herdsmen families. Economic benefits in this study meant whether herdsmen implement GECSRP depended on the trade-off between family costs and gains. Before participating in activities, herdsmen should first consider whether they could obtain considerable benefits (e.g., labor saving, high productivity, high returns) [3,24], as this was more likely to improve their policy satisfaction and implement environmentally friendly activities. Thus, the following hypothesis was proposed:

**Hypothesis 1a (H1a).**  *Economic benefits had positive effects on herdsmen’s GECSRP satisfaction*.

In addition, due to the continuous aggravation of environmental problems, the public had a stronger awareness of the surroundings, and their environmental concepts would affect their satisfaction with their life and production behaviors [25,26]. As one of the main subjects of grassland production activities, the public’s environmental perception could reflect their evaluation of the environment after being destroyed [27]. Although environmental perception had been concerned by some scholars [12,28], few studies had introduced it into the field of ecological grazing to investigate herdsmen’s GECSRP satisfaction. In this study, herdsmen’s environmental value perception meant that whether herdsmen implement GECSRP depended on their consideration of ecological environment. Truelove and Gillis [29] indicated that people’s environmental value was the psychological basis for proenvironment behaviors, and positive perception would improve people’s satisfaction and promote them to actively implement green behaviors. Therefore, the following hypothesis was proposed:

**Hypothesis 1b (H1b).**  *Environmental value had positive effects on herdsmen’s GECSRP satisfaction*.

Sheth et al. [19] emphasized that individual value perception was influenced by social environment in the research of consumer behaviors. When coping with the impacts brought by social environment, social members tended to recognize those who had a strong desire to meet their needs, and realized self-affirmation and promotion by accepting others’ approval [30]. For example, in the field of social media marketing, Chen and Lin [31] found that gaining identity from others could significantly affect the satisfaction of platform users to a great extent. Nevertheless, satisfaction came from measuring the customer’s perception of the company’s product experience, and it also belonged to the individual’s evaluation of their own experience and perception in a way. Vignoles et al. [32] found that social identity was associated with greater satisfaction after exploring the influencing factors of people’s behavioral motivation. For example, villagers living in rural social environment generally paid attention to the evaluation of people around them, which could affect their satisfaction [33]. For herdsmen, social identity was that whether herdsmen implement GECSRP depended on their social recognition. As a consequence, the following hypothesis was proposed:

**Hypothesis 1c (H1c).**  *Social identity had positive effects on herdsmen’s GECSRP satisfaction*.

### 2.2. Environmental Regulation

Broadly speaking, satisfaction was related to a wide range of social variables, including economic, institutional, psychological, and other aspects. As a typical institutional factor, environmental regulation generally referred to the regulatory measures taken by the government to intervene and manage microsubjects in the process of environmental protection, with the purpose of reasonably planning resources and thus promoting sustainable economic development [34,35]. At present, environmental regulation tools had shown a trend of diversified development. Under the condition of information asymmetry, binding regulations combined the advantages of command and competition, and achieved the optimal allocation of environmental resources by reducing the risks brought by adverse selection [36]. Besides, incentive and guiding regulations had been widely applied in the driven social governance with the integration of social governance systems [37]. Therefore, this study set up three dimensions (incentive regulation, guiding regulation and binding regulation) from the dual perspectives of herdsmen’s acceptance and government behaviors to measure environmental regulation based on the relevant research. The following hypothesis was proposed:

**Hypothesis 2 (H2).**  *Environmental regulation had positive effects on herdsmen’s GECSRP satisfaction*.

At present, the research on incentive regulation has been applied and developed in the fields of enterprise operation, medical management and waste disposal. Bian and Fabra [38] indicated that enterprises used market signals to encourage employees to increase their output rate by giving monetary incentives or subsidies. Waddimba et al. [39] found that incentive policies formulated by hospitals could effectively improve the job performance and satisfaction of clinicians. Di Foggia and Beccarello [40] studied the impacts of environmental regulation on waste disposal through the Italy case, and found that incentive regulation had a catalytic effect on waste classification. It could be concluded that incentive regulation had a significant impact on the public’s job satisfaction, which had also been confirmed in the study of [41]. For herdsmen in the surveyed areas, subsidies and rewards were conducive to making up the costs of green production for herdsmen, and ensuring them to obtain corresponding benefits, so as to mobilize the enthusiasm of herdsmen to carry out ecological animal husbandry. Incentive regulation in this study only referred to the impacts of government subsidies and rewards on herdsmen’s ecological animal husbandry. As a means for local government to encourage herdsmen to fulfill grassland ecological protection policies for ecological animal husbandry, it might affect the herdsmen’s satisfaction. Thus, it was necessary for this study to introduce incentive regulation to measure herdsmen’s satisfaction. The following hypothesis was proposed:

**Hypothesis 2a (H2a).**  *Incentive regulation had positive effects on herdsmen’s GECSRP satisfaction*.

According to research by Nicod et al. [42], employees performed better when they were given appropriate capabilities, motivations and organizational opportunities. “Green” training was a very environmentally friendly activity designed to develop interventions with green related capabilities. Pinzone et al. [43], through a survey of employees in the Italian medical sector, found that “green” training could motivate employees to engage in free decision-making related to environmental behaviors, thus making them more satisfied with their jobs and occupational experience. Nicod et al. [42] also highlighted that technical training for employees in the aspect of proenvironment was conducive to improving customer satisfaction. In this study, guiding regulation was the technical publicity measures that the local government implemented to promote the grassland ecological protection policy and thus impact the herdsmen’s ecological animal husbandry. Government’s timely technical training and publicity education could enhance herdsmen’s cognition and understanding of ecological animal husbandry [26], so as to guide herdsmen to adjust their nonecological animal husbandry behaviors. In the field of animal husbandry production, herdsmen were more easily influenced by the rationing mechanism such as education, training and guidance promoted by policy makers, because most of them did not have a deep understanding of the detailed regulations of GECSRP. Therefore, the following hypothesis was proposed:

**Hypothesis 2b (H2b).**  *Guiding regulation had positive effects on herdsmen’s GECSRP satisfaction*.

Besides, the positive effects of legislative constraints on environmental regulatory policies in supervising public behaviors had been demonstrated [44]. Binding regulation could promote the public’s self-policing and strengthen their recognition of green development, and thus improve their satisfaction with regulations [45]. Moreover, it was also very applicable in industrial management, export trade and other fields. Yao et al. [46] showed that binding regulation was an important factor to improve the green production performance and had an important role on industrial development. Ge et al. [47] found that binding regulations were conducive to increasing nation’s export trade earnings through investigating the heterogeneous impacts of environmental laws and financial constraints on China’s green design. Binding regulation in the process of animal husbandry was the supervision and punishment measures made by the local government for herdsmen’s failure to implement the grassland ecological protection policy, which could have an impact on herdsmen’s ecological animal husbandry. The purpose of the government to enact administrative regulations was to directly curb the negative externalities brought by nonecological animal husbandry behaviors, which would also have a significant impact on herdsmen’s satisfaction [48]. Thus, the following hypothesis was proposed:

**Hypothesis 2c (H2c).**  *Binding regulation had positive effects on herdsmen’s GECSRP satisfaction*.

### 2.3. Interaction between Value Perception and Environmental Regulation

The value perception in environment was based on the mutual effect between individuals and the environment [29]. With the aggravation of global environmental changes, environmental pollution not only caused huge economic losses to the society, but also had negative impacts on family livelihood. This point had reached a consensus in the research of many scholars [24,37,49]. Therefore, it was urgent to formulate effective environmental regulations that could promote the sustainable development of social economy.

As the producer and user of grassland resources, herdsmen’s timely response to environmental regulation could prompt them to generate positive environmental perception quickly [34], so as to guide their production behaviors and lead to good changes of production structure. However, the current environmental regulation required herdsmen to adopt ecological animal husbandry mode for sustainable production, which need herdsmen’s ecological consciousness [9]. Therefore, whether to adopt environmental regulation depended largely on the actionability perceived by herdsmen. In fact, herdsmen had different attitudes and values to participate in environmental practices [13]. Government’s environmental regulation was closely related to the value orientation of local public, and solving environmental problems depended on the perception after public participation. Zhang et al. [25] indicated that environmental system not only directly affected life satisfaction, but also indirectly affected it by affecting the environmental quality perception. Environmental value was a kind of value expression of environmental quality. Research viewpoints inspired us to explore the influence of the interaction between environmental regulation and value perception on satisfaction. Moreover, Alcover et al. [36] found that the impacts of environmental regulation on the satisfaction were influenced by customers’ economic benefits perception after exploring the impact mechanism of environmental regulation on customer satisfaction in Portugal’s green enterprises, which was an important dimension of value perception [23]. These research results revealed that there might be a certain interaction between environmental regulation and value perception.

To be specific, guiding regulation guided herdsmen to form positive values, which was conducive to environmental protection by publicizing the importance of grassland environmental protection and the good effects of ecological animal husbandry. While, incentive regulation strengthened the formation of this value by giving economic rewards to herdsmen who fulfilled the ecological livestock husbandry. In contrary, although binding regulation imposed penalties to the behaviors of forbidden grazing, it was actually a forward facilitation of environmental value. In short, environmental regulation would act positively on herdsmen’s value perception of grassland ecological environment. Furthermore, Gu et al. [9] showed that herdsmen were often affected by the behaviors of people around them, that was to say, they were more concerned about others’ recognition of their own behaviors. Environmental regulation also influenced the cultural effect of herdsmen collectivities [50], then their value perception of participating in environmental protection actions, and finally the satisfaction of herdsmen with GECSRP. Therefore, the following hypothesis was proposed:

**Hypothesis 3 (H3).**  *There was an interaction between environmental regulation and value perception, and it had positive effects on herdsmen’s GECSRP satisfaction*.

**Figure 1:**
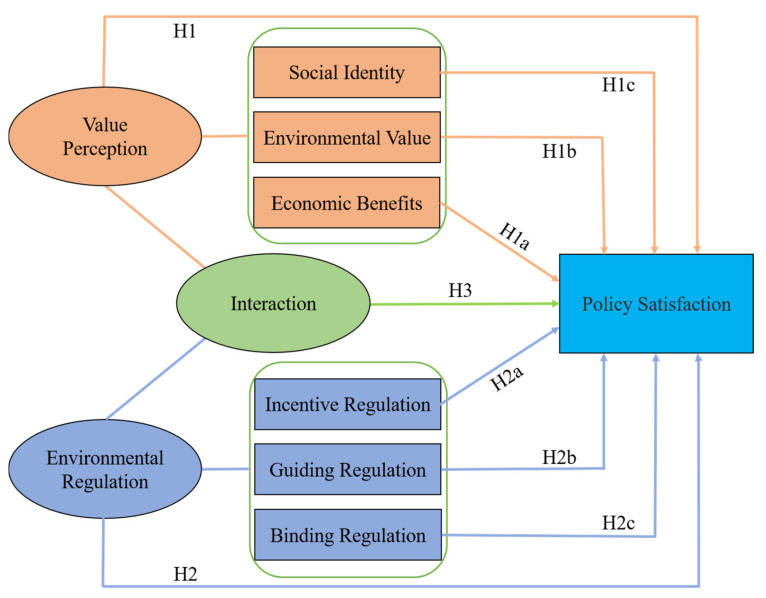
Theoretical analysis framework of this study. H1, H1a, H1b, H1c, H2, H2a, H2b, H2c and H3 indicated the hypotheses proposed in this study.

In view of the above analysis, a theoretical analysis framework as shown in Figure 1 was constructed to verify the above hypotheses in this study. The framework included two core explanatory variables, namely value perception and environmental regulation. It also included an explained variable, namely policy satisfaction. This indicated that value perception and environmental regulation and their interaction could affect herdsmen’s GECSRP satisfaction. Besides, the two-dimensional variables of value perception, namely economic benefits, environmental value and social identity, and the two-dimensional variables of environmental regulation, namely incentive regulation, guiding regulation and binding regulation, also could affect the GECSRP satisfaction.

## 3. Materials and Methods

### 3.1. Study Area and Overview of Subsidy and Reward

The study area was the Inner Mongolia Autonomous Region (Inner Mongolia for short) in North China, which ranged from northeast to southwest with an area of about 1.18 million km^2^, spanning the temperate semihumid, semiarid and arid climate zone [9]. Meanwhile, there were various types of grasslands in this region, including meadow steppe, typical steppe and desert steppe, which made it occupy an important position of animal husbandry in the whole country. Pasture land accounted for about 67% of the total land area of Inner Mongolia, which was the key region concerned by GECSRP.

The GECSRP of this region mainly included three aspects: the subsidies for forbidding grazing, the rewards for balancing grass and livestock, and the production subsidies for herdsmen. In the new round of the plan, the central government arranged CNY 18.76 billion in subsidies and rewards for grassland ecological conservation, where the forbidding grazing was 1.206 billion mu and the balancing between grass and livestock was 2.605 billion mu [51]. According to the investigation, the subsidy standard of forbidden grazing and balancing grass and livestock in Alxa League was determined based on the registered residence age of herdsmen. Hulunbeier city’s subsidy for forbidden grazing was CNY 13.751 per mu per year, and Ulanqab city gave subsidy for forbidden grazing and reward for livestock balance CNY 7.2 and CNY 2.4 per mu per year, respectively. While the subsidy standard of balancing grass and livestock and forbidden grazing in Xilin Gol League was determined according to the grassland area, which was, respectively, CNY 3 and CNY 9 per mu per year. (Note: 1 ha ≈ 15 mu; USD 1 ≈ CNY 6.467).

### 3.2. Sample and Data Collection

The data were derived from the field survey conducted by the research group in the grassland pastoral areas of the western Alxa League, the middle Ulanqab City and Xilin Gol League, the eastern Hulunbeier City from March to July 2020. These areas could better present the situation of animal husbandry production and ecological environment and others in Inner Mongolia. Drawing on the multistage sampling principle of Delle Site et al. [52] and Li et al. [24], a total of nine typical banners (counties) were extracted according to the environment of pastoral areas and the actual situation of herdsmen’s livelihood in this study. Two sapwood (townships) were selected from each county, and 33 herdsmen families from each township were interviewed to obtain their real information. The research group, before the formal survey, went to the pastoral areas around Ordos City for a preliminary test in order to ensure the rationality of the questionnaire design. After that, according to the actual situation of the research areas, three professors and six graduate students from relevant research fields were invited to modify and supplement some interview questions and set answers in the questionnaire, such as production and system characteristics and so on. Finally, after many discussions, this study formed the final formal questionnaire, which involved in value perception, environmental regulation and their dimensions, as well as basic demographic characteristics.

It should be mentioned that most of the interviewees were Mongolian herdsmen who only spoke the local dialect. The research team recruited some people (most of these were local undergraduates or graduate students who could understand the dialect) who could explain the dialect well to assist the face-to-face interview and questionnaire filling. In this way, the biases and errors caused by language differences could be avoided as far as possible. Besides, all investigators were given unified professional training and simulation exercises before the formal survey, so as to avoid the potential bias in answers due to the fact that researchers might be confused with official language. At the end of each interview, bags of milk prepared in advance would be given to the interviewee as a token of appreciation. After the survey was completed, the interview recordings were played repeatedly, and checked with people who could understand the dialect for some questions arising during the interview. Finally, the sorted data would be input timely into a special database according to the actual situation.

**Figure 2:**
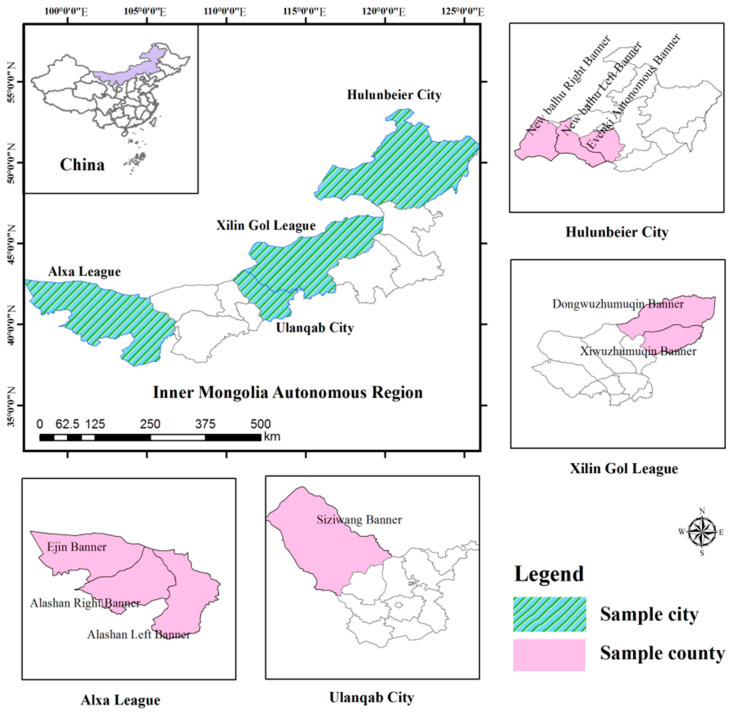
Location of the sample areas in this study.

In this study, a total of 594 questionnaires were issued. After eliminating the invalid and missing values, a total of 562 valid samples were finally obtained, with an effective questionnaire collection rate of 94.61%, among which 143 were from Alxa League, 135 from Xilin Gol League, 165 from Ulanqab City, and 119 from Hulunbeier City. The location of the sample areas is shown in Figure 2. In the Figure 2, three sample counties were selected from Alxa League, including Ejin Banner, Alashan Right Banner and Alashan Left Banner; a sample county, namely Siziwang Banner, was selected from Ulanqab City; three sample counties were selected from Hulunbeier City, including New Balhu Right Banner, New Balhu Left Banner and Evenki Autonomous Banner; two sample counties, namely Dongwuzhumuqin Banner and Xiwuzhumuqin Banner, were selected from Xilin Gol League.

### 3.3. Variables Selection and Measurement

Based on the actual situation of herdsmen and their families in the surveyed areas, this study finally chose herdsmen’s GECSRP satisfaction (hereinafter referred to as “policy satisfaction”) as the explained variable according to the criteria for rigorously measuring explained variables, clearly defining concepts, and accurately collecting data. Likert 5-Point Scale was adopted to accurately reflect the quantitative data of practical problems. Meanwhile, by referring to the research of Kim et al. [53] and Partelow et al. [54], the policy satisfaction was measured by setting the following question: “how is your overall satisfaction with GECSRP”. The scale range was divided into five grades, namely, very dissatisfied, not very satisfied, general, relatively satisfied, and very satisfied, and was assigned as 1, 2, 3, 4, and 5, respectively.

The core explanatory variables were herdsmen’s value perception and environmental regulation. Coteur et al. [55] indicated that herdsmen’s decision-making was not only affected by profitability, but also by available resources and environment [56]. When the benefits were greater than the costs, herdsmen would make more positive evaluations on the results of implementing a certain behavior, and then take the initiative to practical actions. Schulte et al. [57] showed that saving resources and winning social recognition could enhance the endogenous power for individual to implement proenvironmental behaviors. In view of this, three dimensions of economic benefits, environmental value and social identity were adopted to measure the value perception of herdsmen’s GECSRP satisfaction according to existing studies [24,31]. However, most of the existing literature on environmental regulation had stimulated, guided and restrained the public from multiple dimensions. Government’s environmental behavior orientation was a key factor influencing the evaluation of herdsmen’s policy satisfaction, and the acceptance of environmental regulation was also an important reason for herdsmen to carry out animal husbandry production in an ecological way. Therefore, according to the research of Nordlund and Garvill [17], Si et al. [37] and Alcover et al. [36], three dimensions of incentive regulation, guiding regulation and binding regulation were selected to measure environmental regulation from the dual perspectives of herdsmen’s acceptance and government behaviors.

The measurement items of explained variable and core explanatory variables in this study are shown in Table 1.

In addition, according to the research of Gao et al. [3], Wang et al. [6] and Zhang et al. [10], other factors influencing herdsmen’s GECSRP satisfaction were selected as the control variables, including individual characteristics (e.g., gender, age, education level, occupation type), family characteristics (e.g., household labor force, annual income level), production characteristics (e.g., pasture area, whether they have title certificate or not, grassland degradation situation, whether to attend training or not), institution characteristics (e.g., subsidy and award criteria evaluation, whether to pay in time), environmental characteristics (e.g., distance from the supply and marketing market, distance from the livestock sector).

### 3.4. Economic Modeling

The explained variable “policy satisfaction” in this study was a multiclassified ordered variable, which could be investigated by using the multiordered Logit model. Traditional regression models required complete independence between samples. Although it was impossible for samples to be completely independent, it could be solved by using multiordered Logit. Therefore, a multiordered Logit model was selected to analyze herdsmen’s GECSRP satisfaction based on the research of Alcover et al. [36]. The equation structure of the economic model in this study was as follows (Equation (1))

(1) $${Satisfaction}^{*}=\alpha VP_{i}+\beta ER_{i}+\delta X_{i}+\mu_{i}^{*}\;i=1,\;2,\;3,\;4\ldots \ldots n$$

In the above equation, ${Satisfaction}^{*}$ was an unobservable latent variable (herdsmen’s policy satisfaction), $VP_{i}$ was a value perception variable, $ER_{i}$ was an environmental regulation variable, $X_{i}$ was a controlled variable that affects herdsmen’s policy satisfaction, α,β,δ were be estimated coefficients, $\mu_{i}^{*}$ is disturbances term to the standard normal distribution. Satisfaction was the dependent variable with the value range of {1, 2, 3, 4, 5}. The relationship between observable herdsmen’s policy ${Satisfaction}^{*}$ score and unobservable latent variable was as follows (Equation (2)):

(2) $$Satisfaction=\{\begin{array}{c} 1\;(very\;dissatisfied)\;{Satisfaction}^{*}\leq C1\\ 2\;(less\;dissatisfied)\;C1<{Satisfaction}^{*}\leq C2\\ 3\;(general)\;C3<{Satisfaction}^{*}\leq C4\\ 4\;(more\;satisfied)\;C4<{Satisfaction}^{*}\leq C5\\ 5\;(very\;satisfied)\;Satisfaction\geq C5 \end{array}$$

In the above formula, $c_{i}\left(i=1,\;2,\;3,\;4,\;5\right)$ was the threshold value of different satisfaction evaluation grades of GECSRP for herdsmen, which was estimated simultaneously with the parameters and the coefficients to be estimated. Thus, the probability P of different scores of herdsmen’s satisfaction could be written as, respectively (Equations (3)–(7)):

(3) $$P\left(Satisfaction=1\left|X\right.\right)=\Phi \left(c_{1}-\alpha VP_{i}-\beta ER_{i}-\delta X_{i}\right)$$

(4) $$P\left(Satisfaction=2\left|X\right.\right)=\Phi \left(c_{2}-\alpha VP_{i}-\beta ER_{i}-\delta X_{i}\right)-\Phi \left(c_{1}-\alpha VP_{i}-\beta ER_{i}-\delta X_{i}\right)$$

(5) $$P\left(Satisfaction=3\left|X\right.\right)=\Phi \left(c_{3}-\alpha VP_{i}-\beta ER_{i}-\delta X_{i}\right)-\Phi \left(c_{2}-\alpha VP_{i}-\beta ER_{i}-\delta X_{i}\right)$$

(6) $$P\left(Satisfaction=4\left|X\right.\right)=\Phi \left(c_{4}-\alpha VP_{i}-\beta ER_{i}-\delta X_{i}\right)-\Phi \left(c_{3}-\alpha VP_{i}-\beta ER_{i}-\delta X_{i}\right)$$

(7) $$P\left(Satisfaction=5\left|X\right.\right)=\Phi \left(c_{5}-\alpha VP_{i}-\beta ER_{i}-\delta X_{i}\right)-\Phi \left(c_{4}-\alpha VP_{i}-\beta ER_{i}-\delta X_{i}\right)$$

In the above formula, Φ  was the cumulative density function of the standard normal distribution, and the model was estimated using the maximum likelihood method.

### 3.5. Data Processing Software

The questionnaire data were analyzed adopting the social science statistical software package Stata 15.0 (StataCorp LLC, TX, USA). The invalid and missing values in the questionnaire data was firstly deleted. After that, the specific meaning was analyzed by using multiordered Logit regression estimation results. Finally, OLS was employed to test the robustness of the benchmark regression results, and ArcGIS 10.6 (ESRI, CA, USA) and Microsoft Office 2019 (Microsoft, WA, USA) were used to complete the design and production of the charts.

## 4. Results

### 4.1. Demographic Profile of the Subjects

In the sample of 562 herdsmen (see Appendix A), the proportion of males (49.14%) was slightly lower than that of females (50.86%). Most herdsmen (88.79%) were middle-aged and elderly, and the average age of the sample was 49.38 years old. The majority of herdsmen (82.08%) was junior middle school education or below, and only a minority of herdsmen (17.92%) received senior high school education or above. Most of the herdsmen (73.49%) had 1–3 household labor force, and the average number of family workers in the whole sample was 2.91. A minority of herdsmen (9.96%) had an annual income of CNY 80,000 or more, with an average annual income of CNY 47,000. The vast majority of herdsmen (93.21%) had grassland title certificates. Only a small proportion of herdsmen (29.60%) thought that the grassland degradation was not serious, and most herdsmen (70.40%) believed that the grassland degradation was serious. The proportion of herdsmen (79.50%) who thought that the subsidy and award should be paid in time was much higher than that of herdsmen (20.50%) who thought that the subsidy and awards should not be paid in time. In terms of the subsidy and award criteria, most herdsmen (80.98%) believed that the criteria were low, and only a small number of herdsmen (19.02%) considered that the criteria were relatively high.

### 4.2. Herdsmen’s Policy Satisfaction

**Figure 3:**
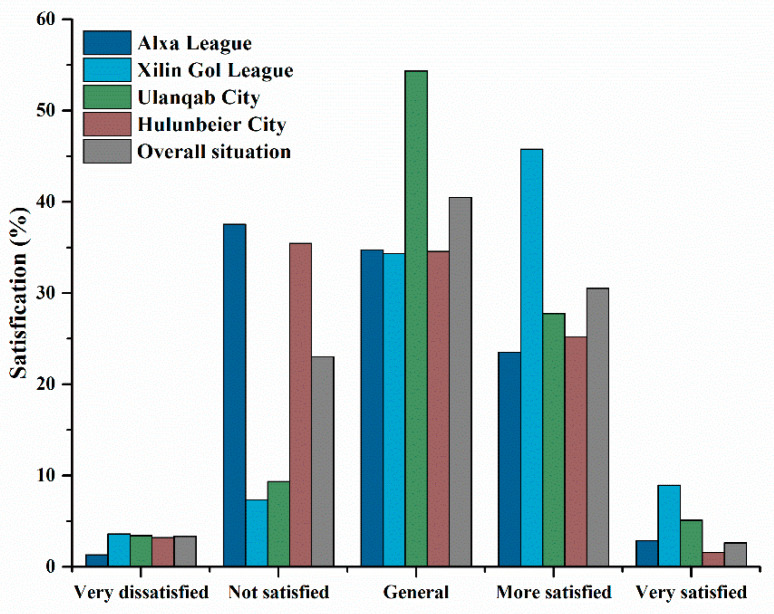
Herdsmen’s satisfaction with GECSRP (*n* = 562). GECSRP is the abbreviation of Grassland Ecological Conservation Subsidy and Reward Policy.

Herdsmen’s policy satisfaction was investigated based on the five-level statement (Figure 3). On the whole, 66.82% of herdsmen rated their satisfaction with GECSRP as the general or below, which indicated that the surveyed herdsmen were not satisfactory with GECSRP. Among them, the proportion of herdsmen in Alxa League and Hulunbeier City who generally rated the GECSRP as general or below was above 70%. Nevertheless, different from these two Leagues (Cities), herdsmen’s policy satisfaction in Xilin Gol League and Ulanqab City was relatively high, those who were satisfied or above accounted for 54.70% and 32.87%, respectively. This indicated that there were obvious regional differences in the evaluation of the herdsmen’s satisfaction with GECSRP. The field investigation found that this might be related to different reward policies and natural environment in the research area. For example, Alxa League’s subsidy standard was based on the age of the household registration, while Xilin Gol League was determined according to the grassland area. Besides, drought, frost and other severe weather occurred frequently in Inner Mongolia, which had obvious impacts on herdsmen who depended on the weather for a living. For example, the growth and quality of grass in Hulunbeier City were good due to sufficient rainfall, and government subsidy had also made up for herders’ spending on buying feed.

### 4.3. Value Perception and Environmental Regulation Variables.

The item “I think GECSRP can bring considerable income to the family” (mean = 3.58) and “I think animal husbandry in an ecological way responds to government policies and wins social recognition” (mean = 3.74) scored relatively high. This indicated that herdsmen attached more importance to economic benefits and social identity in the specific dimension of the variable ‘value perception’ affecting herdsmen’s policy satisfaction. While, most herdsmen (82.45%) scored 3 or below in the item “I think GECSRP has positive significance to the grassland ecological environment”. This meant that the herdsmen tended to “disagree” (mean = 2.56) to a large extent, and their perception of environmental value was not obvious (see Appendix B).

Besides, as for the variable ‘environmental regulation’, the item “The impacts of government subsidy and reward on herdsmen’s ecological animal husbandry” (mean = 3.48) and “The impacts of government technical publicity on herdsmen’s ecological animal husbandry” (mean = 3.50) scored relatively high. This showed that herdsmen’s perception level of the impacts of incentive regulation and guiding regulation formulated by the government on GECSRP satisfaction was moderate, and they relatively recognized the effects of incentive regulation and guiding regulation on the promotion of ecological animal husbandry mode. Nevertheless, the item “The impacts of government supervision and punishment on herdsmen’s ecological animal husbandry” scored relatively low (mean = 2.85), which illustrated that herdsmen had a low degree of approval for binding regulation (see Appendix B).

### 4.4. Baseline Regression Results

Table 2 reveals the baseline regression results. Firstly, the impacts of value perception and environmental regulation on herdsmen’s policy satisfaction were explored separately, and the result was estimated in Model 1. Secondly, six two-dimensional variables including economic benefits, environmental value, social identity and incentive regulation, guiding regulation and binding regulation were introduced to further analyze the impacts of different dimensions on GECSRP satisfaction. The estimated results were shown in Model 2. Thirdly, the interaction term was incorporated to focus on the impacts of interactions of environmental regulation, value perception and their various dimensions on herdsmen’s policy satisfaction, and estimate results were expressed in Models 3 and 4. Finally, the overall fitting effect of each model was good by combining the values of log-likelihood ratio, chi-square and Pseudo R2.

#### 4.4.1. Influence of Value Perception on Policy Satisfaction

Models 1 and 3 showed that value perception (mean) was significant at the level of *p* < 0.001, and the marginal effect was 17.564. Hypothesis H1 was verified. This indicated that the probability of increasing the GECSRP satisfaction would increase by 17.564% with each level increase of the herdsmen’s value perception. Specifically, the estimated results of Models 2 and 4 showed that the two two-dimensional variables, namely economic benefits (*p* < 0.001) and social identity (*p* < 0.01), had significantly positive impacts on the GECSRP satisfaction. Hypotheses H1a and H1c were confirmed. Under the situation that other conditions remained unchanged, if GECSRP could bring some considerable economic income to the family, the probability of herdsmen improving the policy satisfaction increased by 9.735%. Meanwhile, for every level increase of the approval degree of herdsmen to adopt ecological animal husbandry in response to government policies and win social recognition, the probability of their satisfaction with the policies increased by 7.529%. However, environmental value had an insignificantly negative impact on herdsmen’s GECSRP satisfaction, which was contrary to theoretical expectation. Hypothesis H1b was not supported. The field survey revealed that herdsmen’s awareness of ecological and environmental protection was not high enough, and their environmental value perception was slightly inferior compared with material rewards.

#### 4.4.2. Influence of Environmental Regulation on Policy Satisfaction

Models 1 and 3 indicated that environmental regulation (mean) was significant at the level of *p* < 0.001, and the marginal effect was 19.987. Hypothesis H2 was verified. This showed that the probability of herdsmen’s GECSRP satisfaction would increase by 19.987% with each level increase of the impact degree of government environmental regulation. Specifically, the estimated results of Models 2 and 4 showed that incentive regulation and guiding regulation passed the significance test at *p* < 0.001 and hypotheses H2a and H2b were verified. This indicated that both of them had significant and positive effects on the GECSRP satisfaction. When other conditions were controlled, the probability of the herdsmen improving the GECSRP satisfaction would increase by 2.325% for each level increase of the impacts of government subsidy and incentive on their ecological animal husbandry. Moreover, the probability of herdsmen improving their policy satisfaction would increase by 18.758% with each level increase of the impacts of government technical publicity on the ecological animal husbandry. However, binding regulation failed to pass the significance test and hypothesis H2c was not supported. This might be because herdsmen did not approve mandatory regulatory measures, and there existed a phenomenon of “relativity system failure” in the environmental regulation formulated by government, which was not effective in restricting excessive animal husbandry behaviors.

#### 4.4.3. Influence of Interaction on Policy Satisfaction

Model 3 showed that the interaction term between environmental regulation and value perception was significant at *p* < 0.001, and the estimated coefficient was positive. This indicated that environmental regulation could promote the active influence of value perception on herdsmen’s policy satisfaction. There was a certain interaction between environmental regulation and value perception and the direction was positive, so hypothesis H3 was verified.

Furthermore, the specific dimensions of value perception and environmental regulation were introduced and regressed in pairs to further explore the role and relationship between variables. Model 4 showed that the interaction terms of incentive regulation with economic benefits and environmental value were significant at *p* < 0.01, and the estimated coefficients of both were positive. This indicated that incentive regulation could strengthen the positive role of economic benefits and environmental value in improving the policy satisfaction. Besides, the interaction term between guiding regulation and environmental value was significant at *p* < 0.05, and the estimated coefficient was negative, indicating that negative environmental value would weaken the positive impacts of guiding regulation.

#### 4.4.4. Influence of Control Variables on Policy Satisfaction

In the four models in Table 2, some control variables including gender, age, grassland degradation situation, distance from the supply and marketing market, and distance from the livestock sector had significantly negative impacts on the GECSRP satisfaction. Education level, occupation type, household labor force, annual income level, whether they have title certificate or not, whether to attend training or not, and whether to pay in time had significantly positive effects on the satisfaction. Yet the impacts of pasture area and subsidy and award criteria evaluation were not significant.

### 4.5. Robustness Test

The robustness of the baseline regression results in Section 4.4 was tested, drawing on the methods of existing literature [58,59], by randomly selecting a part of samples and replacing the regression model. To be specific, 281 samples were randomly selected from all 562 samples to conduct OLS regression to test the robustness of the above results. According to the comparison between Table 2 and Table 3, the significance of value perception and environmental regulation, as well as two-dimensional variables and interaction terms were relatively consistent among the estimation results of Logit and OLS models, which indicated that the model estimation results had strong robustness.

## 5. Discussion

This study evaluated herdsmen’s GECSRP satisfaction using multiordered Logit model based on the research data of herdsmen in four Leagues (Cities), where they mainly engaged in animal husbandry production in Inner Mongolia of China. Results revealed that herdsmen in the surveyed areas were not satisfied with GECSRP on the whole. To identify the key factors, a theoretical analysis framework was constructed with value perception and environmental regulation as the core explanatory variables. On this basis, the dimensions of these core explanatory variables, namely, economic benefits, environmental value, social identity, incentive regulation, guiding regulation, and binding regulation, were respectively introduced into the research model for further in-depth exploration. Research found that the model had passed the robustness test, and the data analysis results were relatively reliable. Moreover, core explanatory variables (including various dimensions) and control variables had a high fitting goodness in the theoretical model.

In the process of implementing GECSRP, it was essential to note the herdsman’s value perception and how the identified factors influenced the policy satisfaction. Herdsmen, as the key subjects of grassland livestock husbandry, should be the most vocal value feedbacker for the policy, because they could truly and directly perceive whether GECSRP could bring real benefits to people. Results showed that herdsmen’s value perception had direct and positive effects on the policy satisfaction, which was supported by the previous study exploring customer satisfaction [36]. Furthermore, the more economic income GECSRP could bring to the family, the higher of the approval degree of herdsmen to win the respect of the people around them, the more likely it was to improve the herdsmen’s satisfaction with the policy. The possible explanation was that subsidies and rewards for grassland ecological protection had become the second most important income source for some herdsmen’s families [14], and herdsmen’s perception of economic benefits would greatly affect their policy satisfaction. In addition, the herdsmen, due to the generally low education level, did not have a deep understanding of the environmental significance of ecological animal husbandry [49], which resulted in their relatively deficient perception ability of environmental value. Gu et al. [9] pointed out that for the vast majority of herdsmen, economic benefits directly affected their living standard, while the community culture of herdsmen also affected their evaluation of life to a large extent [12,31]. Thus, economic benefits and social identity had greater impacts on the policy satisfaction than herdsmen’s environmental value perception. Hence, it could be predicted that improving herdsmen’s value perception, especially their perception of economic benefits and social identity, was conducive to enhancing herdsmen’s satisfaction with GECSRP.

The influence of environmental regulation on the GECSRP satisfaction was different from the value perception. In particular, the government’s guiding regulation and incentive regulation on ecological grazing had significantly positive effects on the improvement of satisfaction. The more the government paid attention to technical publicity, the more in time it gave certain subsidies and incentives to herdsmen, the more effectively it could improve herdsmen’s satisfaction with GECSRP. This was basically consistent with the research results of Song et al. [60], which emphasized that giving certain tax preference could increase the risk tolerance in research and development. Qian et al. [15] also indicated that reasonable incentive policies could improve farmers’ recognition of green production, and thus enhance their continuous satisfaction with ecological compensation for green fertilizer plans. The government could effectively reduce environmental risks and their negative impacts in the whole production process by recommending producers to implement green production through guiding regulation [61]. These conclusions explained the effectiveness of incentive regulation and guiding regulation in different fields, and also demonstrated the improvement effects of the government’s implementation of these regulations on the GECSRP satisfaction. However, the model results (Table 2) showed that the impacts of binding regulation were not significant. This indicated that most herdsmen believed that government regulatory punishment had little effects on ecological animal husbandry. The reason might be that most herdsmen’s families had engaged in animal husbandry for generations, and they were unwilling to give up or change their inherent grazing ways [9]. In addition, it was difficult for grassland ecological policies to take into account the short-term dynamic needs and aspirations in time, so herdsmen rejected mandatory supervision measures [44].

In addition to the direct effects of value perception and environmental regulation, this study also focused on the interaction between them. Results found that there were significantly positive interactions between environmental regulation and value perception. Wei et al. [62] figured out that external environment could significantly affect farmers’ value perception of edible mushroom cultivation wastes, and value perception had differentiated impacts on farmers’ decision-making behaviors due to different environmental regulations [63]. In terms of the pair-wise interaction between two-dimensional variables, the results of this study showed that the interaction between guiding regulation and environmental value was negatively significant. This was because when the government provided ecological animal husbandry technology training, it often required herdsmen to abandon the original idea and use a new way of ecological animal husbandry, while herdsmen’s inherent environmental value prevented the guiding regulation from playing a positive role. Si et al. [37] indicated that guiding regulation aimed at improving environmental quality and sustainable usage of natural resources by guiding producers to change their production strategies. Most herdsmen reflected that technical training contents were too theoretical, so that they were unable to understand the long-term significance of ecological animal husbandry, nor could they apply the technical knowledge they had been trained to practice. This was not conducive to the formation of positive environmental value, and thus weakened the positive impacts of herdsmen’s environmental value on policy satisfaction to a large extent. This study also found that the interactions of incentive regulation with economic benefits and environmental value were positively significant. Incentive regulation included green production subsidies, financing support and tax relief. The GECSRP aimed to strengthen herdsmen’s perception of economic benefits through issuing subsides and rewards, thus improve herdsmen’s understanding of the significance of grassland ecological environmental protection and guide them to form positive environmental value. Li et al. [24] highlighted that government compensation for farmers adopting ecological protection actions would make them more aware of environmental values, and maximize the transformation of these values into economic benefits, thus enhance the sustainability of ecological compensation.

In this study, the control variables, such as education level, occupation type, household labor force, annual income level and grassland degradation situation and so on, significantly affected herdsmen’s policy satisfaction. The higher educated herdsmen had a stronger awareness of sustainable grazing, so it was easier for them to understand the environmental significance of GECSRP. Gao et al. [3] found that education should not be ignored as a policy tool to promote grassland ecological protection. Besides, families that relied solely on grassland grazing for their livelihood were less able to withstand risks [51], while herdsmen with a variety of occupations were more likely to have other household income sources, and would not be less satisfied with GECSRP due to the lower subsidy. In addition, the issue of pasture title certificate could encourage herdsmen to engage in animal husbandry production for a long time [50], and high-quality pasture could save herdsmen’s cost of buying forage grass. Therefore, the herdsmen families with certificates and better grassland quality were more satisfied with the policy. The study also incorporated geographical environment characteristics as the control variable. Results showed that the closer to the supply and marketing market, the lower the costs of animal husbandry, and the closer to the livestock sector, the easier it was to receive technical training [14], the easier it was also to improve herdsmen’s satisfaction.

The findings of this study provided some policy implications. First, the subsidy and reward model should be adjusted appropriately according to the differences in grassland types, pasture area, production capacity, and other resource endowments in different regions, and the standard of environmental subsidies for herdsmen’s ecological animal husbandry should be increased, especially in terms of basic economic benefits. Second, as herdsmen were easily affected by the grazing patterns of people around them, a model publicity system should be established to appropriately reward herdsmen who followed the policy. Meanwhile, the role of social identity should be strengthened to promote the enthusiasm of herdsmen to protect the ecological environment. Third, the enhanced interaction between environmental regulation and value perception should be coordinated to maximize the effects of publicity, subsidies, regulation, and other activities considering the positive interaction relationship between these information sources. Finally, the social governance platform APP and other approaches could be adopted to widely publicize and explain the GECSRP in depth, so as to improve herdsmen’s policy awareness and environmental value perception, which could further deepen their concept of saving utilization and circular development of grassland resources.

Despite the in-depth exploration, the limitations of this study should be acknowledged. Firstly, the direct impacts on herdsmen’s policy satisfaction were only explored from the perspective of value perception and environmental regulation. Future research could further include other possible mediating factors, such as trust and loyalty. Secondly, different types of herdsmen were not distinguished. Considering that different herdsmen (e.g., pure herdsmen, semi-farmers and semi-herdsmen) had heterogeneity [24], their influence on the satisfaction of GECSRP would also be different, which might be the focus of future research based on predetermined hypotheses. Thirdly, the concept of environmental regulation was defined as external environmental factors, and it mainly referred to the formal system issued by the government related to preserving ecological environment, so as to regulate herdsmen’s ecological animal husbandry activities. Thus, future studies could try to incorporate informal institutions to explore the policy satisfaction. Finally, the research on GECSRP could be extended to other different countries, and some interesting conclusions might be drawn by comparing the GECSRP satisfaction in different countries.

## 6. Conclusions

The purpose of this study was to bring value perception, environmental regulation and their interactions into the unified theoretical analysis framework, so as to deeply explore herdsmen’s GECSRP satisfaction and the key factors behind it. Four typical pastoral areas in Inner Mongolia of China were selected, first-hand data was collected through face-to-face interviews and questionnaires, and a multiordered Logit model was established undertake an empirical analysis. The followings were the main conclusions through the analysis:

(1) Herdsmen were not very satisfied with GECSRP on the whole, while the value perception and environmental regulation variables played a positive role in improving herdsmen’s policy satisfaction. The dimensions of value perception (i.e., economic benefits and social identity) and environmental regulation (i.e., incentive regulation and guiding regulation) had significantly positive effects on the satisfaction. Among them, the economic benefits had the strongest promotion impacts with a marginal effect of 9.735%, followed by the social identity with a marginal effect of 7.529% in the two-dimensional variables of value perception. The guiding regulation had stronger promoting impacts with a marginal effect of 18.758%, followed by the incentive regulation with a marginal effect of 2.325% in the two-dimensional variables of environmental regulation.
(2) Neither the environmental value dimension in the value perception variable nor the binding regulation dimension in the environmental regulation variable had any significant impacts on the GECSRP satisfaction, because herdsmen did not think that the GECSRP would have a greatly positive impact on the grassland ecological environment. Meanwhile, the environmental regulation formulated by government also had a phenomenon of “relative system failure”, which did not have significant impacts on restricting the excessive animal husbandry behaviors.
(3) The interaction term between environmental regulation and value perception had a significant impact on herdsmen’s GECSRP satisfaction. More precisely, incentive regulation played an enhanced interactive impact on the influence of economic benefits and environmental value on the satisfaction. Yet guiding regulation weakened the interaction between herdsmen’s environmental value and their policy satisfaction. This indicated that the technical publicity measures adopted by government were not effective in promoting herdsmen’s policy satisfaction, and the negative environmental value perception would also weaken the positive impacts of guiding regulation.

## Figures and Tables

**Table 1:** Measurement items of variables.

Variables	Dimensions	Items	Scales
Policy satisfaction	—	The herdsmen’s overall satisfaction with GECSRP	1–5: very dissatisfied to very satisfied
Value perception (VP)	Economic benefits VP1	I think GECSRP can bring considerable income to the family	1–5: low to high level of agreement
	Environmental value VP2	I think GECSRP has positive significance to the grassland ecological environment	
	Social identity VP3	I think animal husbandry in an ecological way responds to government policies, and will win social recognition	
	Synthetical value VP4	The arithmetic mean values of VP1, VP2 and VP3 are obtained	—
Environmental regulation (ER)	Incentive regulation ER1	The impacts of government subsidy and reward on herdsmen’s ecological animal husbandry	1–5: low to high level of influencing degree
	Guiding regulation ER2	The impacts of government technical publicity on herdsmen’s ecological animal husbandry	
	Binding regulation ER3	The impacts of government supervision and punishment on herdsmen’s ecological animal husbandry	
	Synthetical value ER4	The arithmetic mean values of VP1, VP2 and VP3 are obtained	—

GECSRP is the abbreviation of Grassland Ecological Conservation Subsidy and Reward Policy.

**Table 2:** Multiordered Logit regression results.

Variables	Model 1	Model 2	Model 3	Model 4	Marginal Effect (%)
Economic benefits		0.531 ***		0.005 ***	9.735
		(2.912)		(0.013)	
Environmental value		−0.015		−0.087	−0.278
		(0.129)		(0.194)	
Social identity		0.416 **		0.558 **	7.529
		(2.540)		(0.991)	
Incentive regulation		0.120 ***		1.658 ***	2.325
		(0.861)		(2.605)	
Guiding regulation		1.030 ***		2.323 ***	18.758
		(5.341)		(3.160)	
binding regulation		0.044		0.219	0.897
		(0.413)		(0.468)	
Incentive regulation * Economic benefits				0.239 **	—
				(2.125)	
Incentive regulation * Environmental value				0.282 **	—
				(2.241)	
Guiding regulation * Environmental value				−0.232 *	—
				(1.863)	
Value perception (mean)	0.877 ***		1.113 ***		17.564
	(4.633)		(1.989)		
Environmental regulation (mean)	0.995 ***		1.231 ***		19.987
	(5.941)		(2.238)		
Environmental regulation (mean) * value perception (mean)			0.071 ***		—
			(0.449)		
Gender	−0.675 **	−0.431 *	−0.686 **	−0.448 *	−8.432
	(2.321)	(1.393)	(2.337)	(1.414)	
Age	−0.019 **	−0.016 *	−0.019 **	−0.016 *	−0.398
	(2.312)	(1.743)	(2.258)	(1.764)	
Education level	0.219 ***	0.205 ***	0.218 ***	0.212 ***	3.986
	(4.059)	(3.591)	(4.049)	(3.654)	
Occupation type	1.281 ***	1.322 ***	1.287 ***	1.436 ***	23.156
	(5.382)	(5.128)	(5.393)	(5.361)	
Household labor force	2.133 ***	2.247 ***	2.112 ***	2.418 ***	41.002
	(3.534)	(3.364)	(3.484)	(3.382)	
Annual income level	0.820 ***	0.870 ***	0.813 ***	0.904 ***	16.785
	(7.062)	(7.183)	(6.941)	(7.055)	
Pasture area	0.007	0.010	0.006	0.006	0.197
	(0.415)	(0.584)	(0.343)	(0.334)	
Whether to have title certificate or not	3.806 ***	3.729 ***	3.813 ***	3.549 ***	28.321
	(5.914)	(5.38)	(5.889)	(5.019)	
Grassland degradation situation	−1.119 ***	−0.922 ***	−1.137***	−0.972 ***	−17.504
	(3.324)	(2.592)	(3.347)	(2.587)	
Whether to attend training or not	1.832 ***	1.616 ***	1.843 ***	1.733 ***	27.761
	(4.906)	(4.067)	(4.904)	(4.224)	
Subsidy and award criteria evaluation	0.072	0.010	0.067	0.007	0.189
	(0.691)	(0.083)	(0.642)	(0.052)	
Whether to pay in a time	3.293 ***	3.115 ***	3.316 ***	3.095 ***	30.751
	(5.671)	(5.163)	(5.671)	(5.054)	
Distance from the supply and marketing market	−0.043 ***	−0.045 ***	−0.043 ***	−0.048 ***	−0.867
	(4.222)	(4.034)	(4.168)	(4.234)	
Distance from the livestock sector	−0.101 ***	−0.097 ***	−0.101 ***	−0.103 ***	−1.987
	(5.759)	(5.094)	(5.768)	(5.249)	
Observations	562	562	562	562	—
Log likelihood	−228.696	−208.959	−228.594	−202.444	—
LR chi2	1087.160	1126.631	1087.361	1139.663	—	
PseudoR2	0.704	0.729	0.704	0.738	—

Only the significant parts were reported here due to space limitation; *, **, and *** was significant at *p* < 0.05, *p* < 0.01, and *p* < 0.001, respectively, the same as below.

**Table 3:** OLS robustness test results.

Variables	Model 5	Model 6	Model 7	Model 8
Economic benefits		0.601 ***		0.002 ***
		(2.642)		(0.031)
Environmental value		−0.013		−0.075
		(0.241)		(0.384)
Social identity		0.463 ***		0.465 **
		(2.861)		(1.021)
Incentive regulation		0.116 ***		1.494 ***
		(0.391)		(2.512)
Guiding regulation		1.049 ***		2.208 ***
		(6.691)		(2.801)
Binding regulation		0.032		0.208
		(0.131)		(0.360)
Value perception (mean)	0.811 ***		1.032 ***	
	(4.218)		(1.562)	
Environmental regulation (mean)	0.933 ***		1.057 ***	
	(5.498)		(1.079)	
Environmental regulation (mean) * Value perception (mean)			0.069 ***	
			(1.600)	
Incentive regulation * Economic benefits				0.214 **
				(2.093)
Incentive regulation * Environmental value				0.275 **
				(2.013)
Guiding regulation * Environmental value				−0.217 *
				(1.104)
Control variables	Controlled	Controlled	Controlled	Controlled
Observations	281	281	281	281
R-squared	0.939	0.944	0.939	0.945

Only the significant parts were reported here due to space limitation; *, **, and *** was significant at *p* < 0.05, *p* < 0.01, and *p* < 0.001, respectively, the same as below.

## Data Availability

The data presented in this study are available on request from the first author.

## Appendix A

**Table A1:** Herdsmen’s demographic profile (*n* = 562).

Variables	Assignment	Frequency	Percentage (100%)	Mean	Standard Deviation
Gender	Male	276	49.14	—	—
	Female	286	50.86		
Age	<30	13	2.31	49.38	9.26
	From 30 to 40	50	8.90		
	From 40 to 50	176	31.32		
	From 50 to 60	315	56.05		
	≥60	8	1.42		
Education level	Primary school and below	219	38.98	—	—
	Junior middle school	242	43.10		
	Senior high school	74	13.20		
	College and above	27	4.72		
Household labor force	From 1 to 3	413	73.49	2.91	0.29
	From 4 to 7	84	14.95		
	>7	65	11.56		
Annual household income (wan yuan)	<2	89	15.84	4.70	1.28
	From 2 to 5	221	39.32		
	From 5 to 8	196	34.88		
	≥8	56	9.96		
Whether to have title certificate or not	Yes	524	93.21	—	—
	No	38	6.79		
Grassland degradation situation	Not serious	166	29.60	1.77	0.42
	Serious	396	70.40		
Whether to attend training or not	Yes	346	61.50	—	—
	No	216	38.50		
Whether to pay in time	Yes	447	79.50	—	—
	No	115	20.50		
Subsidy and award criteria evaluation	Very low	75	13.35	2.99	1.57
	Relatively low	341	60.65		
	General	39	6.98		
	Relatively high	88	15.67		
	Very high	19	3.35		

1 ha ≈ 15 mu; USD 1 ≈ CNY 6.467.

## Appendix B

**Table A2:** Levels of value perception and environmental regulation (*n* = 562).

Variables	Dimensions	Assignment	Frequency	Percentage (100%)	Mean	Standard Deviation
Value perception	Economic benefits	1	13	2.31	3.58	1.51
		2	69	12.27		
		3	195	34.65		
		4	149	26.54		
		5	136	24.23		
	Environmental value	1	159	28.23	2.56	1.21
		2	121	21.57		
		3	183	32.65		
		4	10	1.79		
		5	89	15.76		
	Social identity	1	14	2.43	3.74	1.51
		2	69	12.34		
		3	151	26.78		
		4	145	25.80		
		5	183	32.65		
Environmental regulation	Incentive regulation	1	53	9.43	3.48	1.38
		2	41	7.35		
		3	189	33.56		
		4	143	25.40		
		5	136	24.26		
	Guiding regulation	1	53	9.43	3.50	1.45
		2	30	5.32		
		3	200	35.67		
		4	142	25.32		
		5	136	24.26		
	Binding regulation	1	22	3.87	2.85	1.51
		2	279	49.59		
		3	30	5.36		
		4	225	39.95		
		5	7	1.23		

1–5 presented herdsmen’s agreement degree for a specific dimension that effectively promoted ecological animal husbandry.
